# A case-control and cohort study to determine the relationship between ethnic background and severe COVID-19

**DOI:** 10.1016/j.eclinm.2020.100574

**Published:** 2020-10-09

**Authors:** Rosita Zakeri, Rebecca Bendayan, Mark Ashworth, Daniel M. Bean, Hiten Dodhia, Stevo Durbaba, Kevin O'Gallagher, Claire Palmer, Vasa Curcin, Elizabeth Aitken, William Bernal, Richard D. Barker, Sam Norton, Martin Gulliford, James T.H. Teo, James Galloway, Richard J.B. Dobson, Ajay M. Shah

**Affiliations:** a School of Cardiovascular Medicine and Sciences, James Black Centre, King's College London British Heart Foundation Centre, 125 Coldharbour Lane, London SE5 9NU, UK; b Department of Biostatistics and Health Informatics, Institute of Psychiatry, Psychology and Neuroscience, King's College London, UK; c NIHR Biomedical Research Centre at South London and Maudsley NHS Foundation Trust and King's College London, UK; d School of Population Health and Environmental Sciences, King's College London, UK; e King's College Hospital NHS Foundation Trust, London, UK; f Lewisham and Greenwich NHS Trust, London, UK; g Centre for Rheumatic Disease, School of Immunology and Microbial Sciences, King's College London, UK; h Health Data Research UK London, Institute of Health Informatics, University College London, UK.

**Keywords:** COVID-19, Ethnicity, Comorbidities, Case-control study, Deprivation

## Abstract

**Background:**

People of minority ethnic backgrounds may be disproportionately affected by severe COVID-19. Whether this relates to increased infection risk, more severe disease progression, or worse in-hospital survival is unknown. The contribution of comorbidities or socioeconomic deprivation to ethnic patterning of outcomes is also unclear.

**Methods:**

We conducted a case-control and a cohort study in an inner city primary and secondary care setting to examine whether ethnic background affects the risk of hospital admission with severe COVID-19 and/or in-hospital mortality. Inner city adult residents admitted to hospital with confirmed COVID-19 (*n* = 872 cases) were compared with 3,488 matched controls randomly sampled from a primary healthcare database comprising 344,083 people residing in the same region. For the cohort study, we studied 1827 adults consecutively admitted with COVID-19. The primary exposure variable was self-defined ethnicity. Analyses were adjusted for socio-demographic and clinical variables.

**Findings:**

The 872 cases comprised 48.1% Black, 33.7% White, 12.6% Mixed/Other and 5.6% Asian patients. In conditional logistic regression analyses, Black and Mixed/Other ethnicity were associated with higher admission risk than white (OR 3.12 [95% CI 2.63–3.71] and 2.97 [2.30–3.85] respectively). Adjustment for comorbidities and deprivation modestly attenuated the association (OR 2.24 [1.83–2.74] for Black, 2.70 [2.03–3.59] for Mixed/Other). Asian ethnicity was not associated with higher admission risk (adjusted OR 1.01 [0.70–1.46]). In the cohort study of 1827 patients, 455 (28.9%) died over a median (IQR) of 8 (4–16) days. Age and male sex, but not Black (adjusted HR 1.06 [0.82–1.37]) or Mixed/Other ethnicity (adjusted HR 0.72 [0.47–1.10]), were associated with in-hospital mortality. Asian ethnicity was associated with higher in-hospital mortality but with a large confidence interval (adjusted HR 1.71 [1.15–2.56]).

**Interpretation:**

Black and Mixed ethnicity are independently associated with greater admission risk with COVID-19 and may be risk factors for development of severe disease, but do not affect in-hospital mortality risk. Comorbidities and socioeconomic factors only partly account for this and additional ethnicity-related factors may play a large role. The impact of COVID-19 may be different in Asians.

**Funding:**

British Heart Foundation; the National Institute for Health Research; Health Data Research UK.

**Research in context**

**Evidence before this study**

Minority ethnic groups are reported to experience a higher burden of severe COVID-19 than white individuals but there is uncertainty about the underlying factors and when during the disease trajectory the risk lies. Previous studies adjusted for clinical and demographic factors but involved aggregate analyses over large geographical regions and did not control for the wide local variations in ethnic composition and socio-demographic factors within such regions. A search of PubMed, MEDLINE, preprint and grey literature identified no case-control studies that addressed this question.

**Added value of this study**

This case-control study assessed the association between ethnicity and risk of severe COVID-19 in an ethnically diverse inner city location, taking into account the local contextual population demography and individual-level comorbidity burden and socioeconomic deprivation. We found a strong association between Black or Mixed ethnicity (but not Asian ethnicity) and an increased risk of admission for COVID-19, which was only partly attenuated after adjustment for comorbidities and socioeconomic deprivation. Neither Black nor Mixed ethnicity were independently associated with increased in-hospital mortality risk but a higher in-hospital mortality risk was estimated for Asian patients.

**Implications of all the available evidence**

Individuals of Black and Mixed ethnicity have a high burden of disease because they are disproportionately likely to develop severe COVID-19 requiring admission but they do not have an increased in-hospital mortality at individual level. This contrasts to Asians who may have a worse in-hospital outcome but the admission risk itself does not appear to be higher. A substantial component of the increased risk in both settings remains after adjusting for deprivation measures and comorbidities, suggesting that additional ethnicity-related factors are important.

Alt-text: Unlabelled box

## 1 Introduction

SARS-CoV2 (Severe acute respiratory syndrome coronavirus 2) is a highly transmissible respiratory pathogen that usually causes minor illness but in a small proportion of individuals leads to severe systemic disease (Coronavirus disease 2019 [COVID-19]), with 20–30% in-hospital mortality [1], [2], [3], [4]. Older people, males, and those with comorbidities such as diabetes and cardiovascular disorders are over-represented among those requiring hospital admission [1], [2], [3], [4]. After COVID-19 spread to multi-ethnic populations in Western Europe and North America, numerous reports suggested a higher disease burden in Black, Asian or other minority ethnic groups [5], [6], [7], [8], [9], [10]. Audit data on patients admitted to UK intensive care units (ICU) with COVID-19 observed a substantially higher proportion of minority ethnic background patients than previous years [7]. US data reported higher per-capita mortality rates for Black and Hispanic compared to White people in several cities or in aggregate analyses across large states, but the underlying reasons are unclear [6,9]. A UK Office of National Statistics (ONS) analysis suggested that individuals of Black and South Asian descent had a higher likelihood of death than White people after adjustment for demographic and socioeconomic factors, but was limited by lack of information on comorbidities and the use of historic (2011) data for the reference population [8]. A recent UK nationwide cohort study [11] also reports increased overall mortality in Black and South Asian compared to White people but, importantly, does not take into account the large variations in the ethnic composition of local populations within different geographical regions.

Higher mortality in minority ethnic groups could simply be because minority populations in most Western countries are typically more concentrated in large cities, and these are the very regions that have been most affected by COVID-19. In the UK's largest city, London, 27 of the 33 Boroughs have an ethnic minority population of at least 25% [12]. Furthermore, nine London Boroughs with a high ethnic minority population are among the ten local authorities with the highest age-standardised COVID-19 mortality rates in the UK [12,13]. Many of these regions are also characterised by a higher community prevalence of comorbidities and greater socioeconomic deprivation. Detailed data on socio-demography and comorbidities at local community level are therefore essential to dissect the complex relationship between ethnicity and COVID-19, ideally in a case-control study design to reduce selection bias. This has not been attempted in the studies reported to date nor have they defined when in the disease trajectory ethnicity-related differences are manifest; i.e. infection, disease progression leading to hospitalisation, or in-hospital survival.

We addressed these questions in a London region with approximately 40% Black and minority ethnic background people among a population of 1.26 million. Our aims were to (1) determine the relationship between ethnicity, local population demography, individual-level comorbidities, socioeconomic profiles, and hospital admission for severe COVID-19; (2) establish whether ethnicity is associated with in-hospital outcome of severe COVID-19.

## 2 Methods

### 2.1 Study design and participants

We conducted an observational cohort study at King's College Hospital Foundation Trust (KCHFT), which comprises two separate hospitals in south London. We included consecutive adult patients (age ≥18 years) requiring emergency hospital admission with a primary diagnosis of COVID-19, between 1 March and 2 June 2020. All patients tested positive for viral RNA by quantitative RT-PCR in nasopharyngeal and oropharyngeal swabs.

We performed a case-control (case-population [14]) study with the subset of admitted patients who were inner city residents (>55% of the total hospital cohort). COVID-19 patients (i.e. cases) were matched with population controls sampled from the same inner city region using a primary healthcare database (Lambeth DataNet). This comprises de-identified data on 344,083 (96.8%) community-resident adults registered with 41 practices in inner south-east London [15]. We randomly sampled four controls for each case, individually matched by age (within 5-year age bands) and sex.

### 2.2 Data sources and processing

Demographic and clinical data for admitted patients were retrieved from the electronic health record (EHR). We used well-validated natural language processing (NLP) informatics tools belonging to the CogStack ecosystem to access both structured fields and unstructured text in the EHR [16], [17], [18]. Additional manual extraction, clinician case review, and mandatory hospital datasets were used for missing variables and validation. For population controls, individual-level anonymised Read-coded data were extracted from the structured fields of the primary care EHR database.

### 2.3 Exposures

The primary exposure variable was self-reported ethnicity according to the 18 categories recommended by the ONS [19]. These were reduced into four groups: White (British, Irish, Gypsy, any other White), Black (African, Caribbean, any other Black), Asian (Indian, Pakistani, Bangladeshi, Chinese, any other Asian), and Mixed/Other. Patients with missing ethnicity data were excluded. Demographic and clinical variables, identified a priori as potential risk factors for severe COVID-19, included: age, sex, body mass index (BMI), cardiometabolic comorbidities (hypertension, coronary heart disease [CHD], heart failure, previous stroke or transient ischaemic attack [TIA], diabetes, chronic kidney disease [CKD]), asthma and chronic obstructive pulmonary disease (COPD). For BMI, the most recent value within 6 months (median 27 days [LQ-UQ 0–38]) of admission (for hospital cases) or data extraction (primary care) was used. BMI categories were defined as underweight (<18.5 kg/m^2^), normal (18.5–24.9 kg/m^2^ [18.5–22.9 kg/m^2^ for Asians]), overweight (25–29.9 kg/m^2^ [23–27.4 kg/m^2^ for Asians]), and obese (≥30 kg/m^2^ [≥27.5 kg/m^2^ for Asians]) [20]. Comorbidities were categorised as present if recorded at any time in the EHR up to and including the day of admission (or data extraction in primary care). Socioeconomic status was estimated using the English Indices of Multiple Deprivation (IMD) score, presented in quintiles, as derived from each individual's residential postcode and the relevant 2019 Low Super Output Area (LSOA) code [21]. The IMD score includes data on income, employment, crime, living environment, education and barriers to services. Higher quintiles indicate less deprivation. Disease severity at admission was estimated from the routinely recorded NEWS2 score (National Early Warning Score for degree of illness). Date of self-reported symptom onset was extracted where available. The same variable definitions were used across primary and secondary care.

### 2.4 Outcomes

Outcomes included hospital admission for COVID-19 and in-hospital mortality. The secondary outcome of ICU admission was evaluated in the hospital cohort. Start of follow-up for all analyses was taken as the admission date. Outcomes were ascertained to 2 June 2020.

### 2.5 Statistical analysis

Patient characteristics were summarised using frequency (%) and median with interquartile range (IQR). Comparisons across ethnic groups were made using the χ2 or Fisher's exact test for categorical variables and 1-way ANOVA or Kruskal-Wallis test as appropriate for continuous variables. Where individual comparisons were made, e.g. between Black and White ethnicity, the Bonferroni correction was used. To evaluate the association between ethnicity and hospital admission for COVID-19, we fitted conditional logistic regression models. All models were adjusted for the matching variables (age and sex) to eliminate residual confounding [22] and based on their previously reported associations with COVID-19. Successive adjusted models included adjustment for IMD, cardiometabolic comorbidities, all comorbidities (cardiometabolic comorbidities plus asthma and chronic obstructive pulmonary disease), and finally a combination of all variables (fully adjusted model). Variables were selected based on previous literature and clinical relevance. Age was modelled as a categorical variable to allow for potentially non-linear association with outcomes (18–24, 25–34, 35–44, 45–54, 55–64, 65–74, 75–84, 85+ years). Comorbidities were modelled as binary variables and IMD was fitted as a categorical variable (quintiles). White ethnicity was used as the reference group.

In the hospital cohort, we evaluated the association between ethnicity and risk of in-hospital death using Cox proportional hazards models, with in-hospital mortality as the dependent variable and admission date as the start of the observation window. For the secondary outcome of ICU admission, we used competing risks regression, based on Fine and Gray's proportional sub-hazards model [23], with ICU admission as the dependent variable and death or discharge from hospital assigned as a competing risk. Univariable, age- and sex-adjusted, and fully adjusted multivariable models were performed as for the case-control study with White ethnicity as the reference group. Patients were considered to be right-censored if they were a) discharged from hospital alive, or b) currently in hospital at the study end date. The proportional hazard assumption was examined graphically and using formal tests, using the methods described by Grambsch [24]. No major deviations from this assumption were observed. Analyses were performed using STATA/IC (v16.1; StataCorp LLC, TX). As this study was descriptive, formal power calculations were not performed; however, sample size considerations are highlighted in the Supplemental Methods.

### 2.6 Sensitivity analyses

Since BMI was missing in >30% of hospitalised patients, the primary analyses were performed without adjustment for BMI. To explore confounding by BMI, all analyses were repeated in the subset of patients with BMI data available on a complete case analysis basis, with BMI modelled as a continuous variable. To investigate potential confounding due to differences in timing of hospital presentation between ethnic groups we performed sensitivity analyses using the date of self-reported symptom-onset as the beginning of the observation window, in lieu of hospital admission date, in the subset of patients where this was reported. Additional sensitivity analyses were performed in patients ≥65 years of age, and with imputation of missing comorbidity and IMD variables (<5%) using multiple imputations by chain equations [25].

### 2.7 Ethics

The study adhered to the principles of the UK Data Protection Act 2018, UK National Health Service (NHS) information governance requirements, and the Declaration of Helsinki. De-identified data from patients admitted to KCHFT were analysed under London SE Research Ethics Committee approval (reference 18/LO/2048) granted to the King's Electronic Records Research Interface (KERRI). Specific work on COVID19 was approved by the KERRI committee which included patients and the Caldicott Guardian. Access to Lambeth DataNet was under a project-specific approval granted by Lambeth Public Health Caldicott Guardian; additional informed consent was not required.

### 2.8 Data availability

The corresponding author had full access to all the data and final responsibility for the decision to submit for publication. The data underlying this article cannot be shared publicly due to patient confidentiality regulations. However, we are happy to share the analytical methods that were employed, upon request to the corresponding author.

### 2.9 Role of funding

The funders had no role in study design, data collection and analysis, decision to publish, or preparation of the manuscript.

## 3 Results

### 3.1 Relationship between COVID-19 admission and sociodemographic and comorbidity profiles

**Fig. 1:**
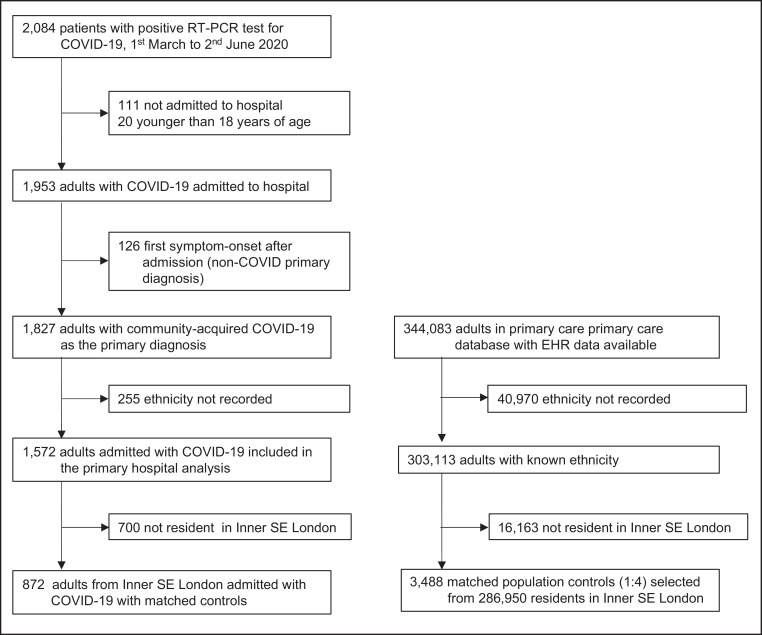
Study flow chart.

Between March 1 and June 2, 2020, 1827 adults were admitted with laboratory-confirmed COVID-19, among whom 872 were inner city residents with ethnicity reported (Fig. 1). We compared this group with the population of adult residents registered with local primary care practices in the same region with ethnicity data available. Population-based patients with no ethnicity reported were marginally younger and had slightly lower rates of comorbidity (Supplemental Table 1).

COVID-19 patients were older (median age [IQR] 66 [55–80] versus 38 [29–51] years; *p*<0•001) and more likely to be male (55•9% versus 49•2%, *p*<0.001) than community residents, across all ethnic groups. However, there was a particularly high rate of admissions among Black people aged 45–65 years, as compared to the community age structure (Supplemental Fig. 1). 65% of patients were from the lowest two quintiles of deprivation as compared to the 52% population prevalence.

To examine the association between ethnicity and hospital admission, each patient with COVID-19 (*n* = 872 cases) was matched by age and sex to 4 randomly sampled population controls (total *n* = 3488, Fig. 1). Cases were more likely than controls to be of Black or Mixed/Other ethnicity and from the lowest deprivation quintile (Table 1). Cases also had a greater prevalence of cardiometabolic morbidities (notably hypertension, diabetes, CKD and CHD), overall (Table 1) and within ethnic groups (Supplemental Table 2).

**Table 1:** Characteristics of COVID-19 cases and matched population controls.

	COVID-19		
	CASES (*N* = 872)	MATCHED POPULATION CONTROLS (*N* = 3488)	P-VALUE
**Demographics**			
Age, years	66 (55–80)	67 (55–79)	–
Age group, n (%)			
18–24	13 (1.5)	52 (1.5)	–
25–34	33 (3.8)	132 (3.8)	–
35–44	47 (5.4)	188 (5.4)	–
45–54	124 (14.2)	496 (14.2)	–
55–64	181 (20.8)	724 (20.8)	–
65–74	160 (18.4)	640 (18.4)	–
75–84	191 (21.9)	764 (21.9)	–
85+	123 (14.1)	492 (14.1)	–
Male sex	487 (55.9)	1948 (55.9)	–
Ethnicity			<0.001
White	294 (33.7)	2019 (57.9)	
Black	419 (48.1)	944 (27.1)	
Asian	49 (5.6)	273 (7.8)	
Mixed/Other	110 (12.6)	252 (7.2)	
**Comorbidities**			
Cardiovascular			
Hypertension	594 (68.1)	1461 (41.9)	<0.001
Coronary heart disease	149 (17.1)	275 (7.9)	<0.001
Heart failure	137 (15.7)	123 (3.5)	<0.001
Previous stroke/TIA	146 (16.7)	213 (6.1)	<0.001
Metabolic			
Diabetes	389 (44.6)	669 (19.2)	<0.001
Chronic kidney disease	228 (26.2)	380 (10.9)	<0.001
Other			
Asthma	123 (14.1)	243 (7.0)	<0.001
COPD	103 (11.8)	200 (5.7)	<0.001
**Socioeconomic factors**			
Deprivation quintile			<0.001
1 (most deprived)	255 (29.2)	678 (19.4)	
2	392 (45.0)	1574 (45.1)	
3	177 (20.3)	953 (27.3)	
4	43 (4.9)	236 (6.8)	
5 (least deprived)	3 (0.3)	31 (0.9)	
Missing	2 (0.2)	16 (0.46)	

Data are presented as n (%) or median (IQR) as appropriate.

**Fig. 2:**
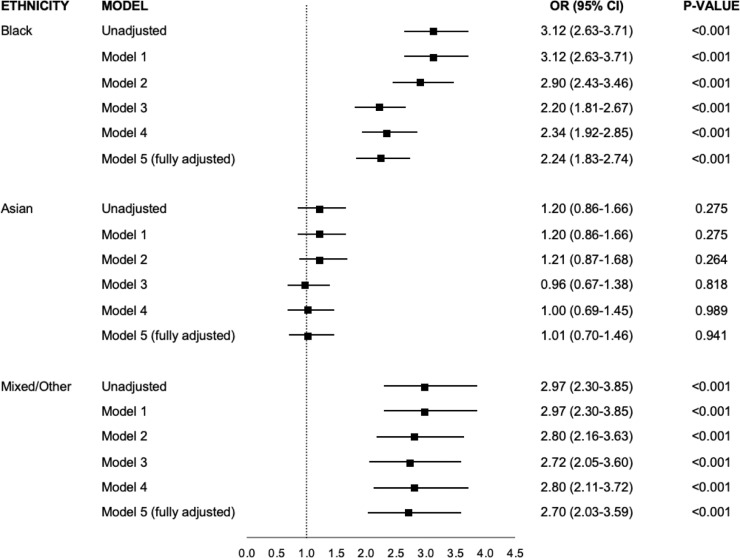
**Association between ethnicity and risk of hospital admission for COVID-19**. Odds ratios are compared to White ethnicity Model 1 – adjusted for age and sex Model 2 – adjusted for age, sex and index of multiple deprivation Model 3 – adjusted for age, sex and cardiometabolic comorbidities* Model 4 – adjusted for age, sex, and all comorbidities** Model 5 (fully adjusted model) – adjusted for age, sex, index of multiple deprivation, and all comorbidities *Cardiometabolic comorbidities include hypertension, coronary heart disease, heart failure, previous stroke/TIA, diabetes, chronic kidney disease. **Cardiometabolic comorbidities, asthma, chronic obstructive pulmonary disease.

In analyses adjusting for the matching variables (age and sex) only, Black and Mixed/Other ethnicity were associated with higher odds of admission compared to White ethnicity (Fig. 2) (OR [95% CI] for Black ethnicity 3.1 [2.6–3.7], Mixed/Other 3.0 [2.3–3.9]; both *p*<0.001). All comorbidities assessed were also associated with greater odds of admission, with the highest OR observed for cardiometabolic comorbidities, including hypertension (4.2 [3.5–5.1]) and diabetes (3.7 [3.1–4.4]). In fully adjusted models including all comorbidities and deprivation quintile, there was modest attenuation of the association between Black or Mixed/Other ethnicity and risk of admission, with the OR still 2.2 to 2.7-fold higher for these groups (Fig. 2). There was no increase in admission risk associated with Asian ethnicity albeit the proportion of Asian patients in our study was small (5.6% cases, 7.8% controls, predominantly non-East Asian). Similar results were obtained in analyses adjusting for BMI, in the subset of individuals with BMI data available. BMI contributed to a small proportion of ethnicity-associated admission risk (Supplemental Fig. 2). An increased OR for admission was observed for both Black African and Black Caribbean groups in disaggregated analyses (Supplemental Table 3).

To assess whether the increased admission risk was specific for COVID-19, we examined all admissions from inner south-east London residents to the 5 main hospitals serving the region, both for COVID-19 (*n* = 2434 patients; March 1 to June 2, 2020) and for respiratory infections over the preceding 12 months (*n* = 7081) (Supplemental Fig. 3). These data confirmed a disproportionately higher number of minority ethnic group patients among those admitted for COVID-19 compared with emergency respiratory infection admissions in the preceding year.

### 3.2 Clinical presentation of COVID-19 in patients requiring hospital admission

Among 1827 patients admitted to KCHFT with COVID-19 from either inner city or suburbs, 1572 had ethnicity data available (Fig. 1). Patients without ethnicity data available were, on average, younger and had fewer comorbidities, but a similar distribution of IMD (Supplemental Table 4).

In patients with ethnicity reported, patients of Black or other minority ethnic background (46.3%) were statistically significantly younger than White patients (median age [IQR]: 61 [51–76] versus 76 [63–86], *p*<0.001) but with a similar sex distribution (male sex: 56.4% minority groups versus 56.3% White) - Table 2. This difference in age profile was sustained over the study period (Supplemental Table 5). Black patients had higher prevalence of hypertension, diabetes and CKD, and a higher proportion of overweight and obese individuals, than White patients. Conversely, Black patients had the lowest rates of CHD and COPD. Asian patients had higher prevalence of diabetes and obesity compared to White and a lower prevalence of COPD. 74% of Black patients resided in localities with the two lowest IMD quintiles as compared to 51% of Asian and 38% of White patients (*p*<0.001).

**Table 2:** Baseline characteristics for patients admitted to hospital with COVID-19 by ethnic group.

CHARACTERISTIC	TOTAL	WHITE	BLACK	ASIAN	MIXED / OTHER
	*N* = 1572	*N* = 845 (53.8%)	*N* = 486 (30.9%)	*N* = 90 (5.7%)	*N* = 151 (9.6%)
**Demographics**					
Age, y	70 (56–82)	76 (63–86)	61 (52–76)	61 (44–78)	60 (49–78)
Male sex	886 (56.4)	476 (56.3)	269 (55.4)	52 (57.8)	89 (58.9)
BMI*, kg/m^2^	26.7 (22.7–31.9)	25.4 (22–30)	28.6 (25–34)	27.3 (25.6–30.7)	27.5 (22.6–31.1)
Underweight	66 (4.2)	52 (6.2)	6 (1.2)	2 (2.2)	6 (4.0)
Normal weight	337 (21.4)	241 (28.5)	64 (13.2)	8 (8.9)	24 (15.9)
Overweight	292 (18.6)	159 (18.8)	95 (19.5)	15 (16.7)	23 (15.2)
Obese	340 (21.6)	155 (18.3)	130 (26.7)	24 (26.7)	31 (20.5)
Missing	537 (34.2)	238 (28.2)	191 (39.3)	41 (45.6)	67 (44.3)
**Comorbidities**					
Cardiovascular					
Hypertension	1027 (65.3)	555 (65.7)	348 (71.6)	47 (52.2)	77 (51.0)
Coronary heart disease	288 (18.3)	187 (22.1)	62 (12.8)	17 (18.9)	22 (18.3)
Heart failure	250 (15.9)	147 (17.4)	71 (14.6)	15 (16.7)	17 (11.3)
Stroke	244 (15.5)	152 (18.0)	73 (15.0)	9 (10.0)	10 (6.6)
Metabolic					
Diabetes	594 (37.8)	242 (28.6)	249 (51.2)	42 (46.7)	61 (40.4)
Chronic kidney disease	405 (25.8)	218 (25.8)	146 (30.0)	17 (18.9)	24 (15.9)
Other					
Asthma	193 (12.3)	87 (10.3)	70 (14.4)	13 (14.4)	23 (15.2)
COPD	202 (12.9)	152 (18.0)	30 (6.2)	6 (6.7)	14 (9.3)
Number of comorbidities (excluding obesity)					
None	346 (22.0)	190 (22.5)	81 (16.7)	23 (25.6)	52 (34.4)
1	376 (23.9)	207 (24.5)	115 (23.7)	24 (26.7)	30 (19.9)
2+	850 (54.1)	448 (53.0)	290 (59.7)	43 (47.8)	69 (45.7)
**Socioeconomic factors**					
Deprivation quintile					
1 (most deprived)	331 (24.2)	117 (13.8)	161 (33.1)	17 (18.9)	36 (23.8)
2	487 (31.0)	200 (23.7)	199 (40.9)	29 (32.2)	59 (39.1)
3	280 (17.8)	140 (16.6)	89 (18.3)	17 (18.9)	34 (22.5)
4	239 (15.2)	195 (23.1)	21 (4.3)	10 (11.1)	13 (8.6)
5 (least deprived)	225 (14.3)	189 (22.4)	12 (2.5)	17 (18.9)	7 (4.6)
Missing	10 (0.6)	4 (0.4)	4 (0.8)	–	2 (1.3)

Data are presented as number (%) or median (IQR).

⁎ BMI was categorised as underweight (<18.5 kg/m^2^), normal (18.5–24.9 kg/m^2^ [18.5–22.9 kg/m^2^ for Asians]), overweight (25–29.9 kg/m^2^ [23–27.4 kg/m^2^ for Asians]), and obese (≥30 kg/m^2^ [≥27.5 kg/m^2^ for Asians]).

Among patients with a recorded date for symptom onset (73.2%), symptom duration before admission was slightly longer in minority ethnic groups (Table 3). However, duration of symptoms was similar across IMD quintiles (median 4 days for both the most and least deprived quintiles). The NEWS2 score at presentation was slightly higher in Black versus White patients (*p*<0.001) but not statistically significantly different in other minority ethnic groups (Table 3).

**Table 3:** Duration of symptoms before admission and in-hospital outcomes for patients admitted with COVID-19 by ethnic group.

	TOTAL	WHITE	BLACK	ASIAN	MIXED / OTHER
	*N* = 1572	*N* = 845 (53.8%)	*N* = 486 (30.9%)	*N* = 90 (5.7%)	*N* = 151 (9.6%)
**Duration of symptoms prior to admission, days**	4 (1–7)	3 (1–7)	4 (2–7)	7 (3–10)	5 (2–7)
Missing	421 (26.8)	265 (31.4)	102 (12.1)	19 (21.1)	35 (23.2)
NEWS2 score*	3 (1–5)	2 (1–4)	3 (2–5)	3 (2–5)	3 (1–4)
Missing	229 (15.0)	104 (12.3)	78 (16.0)	17 (18.9)	30 (19.9)
**Outcomes**					
Died in hospital	455 (28.9)	285 (33.7)	115 (23.7)	30 (33.3)	25 (16.7)
Admitted to ICU	225 (14.3)	90 (10.7)	81 (16.7)	25 (27.8)	29 (19.2)
Death or ICU admission	599 (38.1)	336 (39.8)	169 (34.8)	47 (52.2)	47 (31.1)
Discharged from hospital alive	1046 (66.5)	529 (62.6)	347 (71.4)	57 (65.3)	113 (74.8)

Data reported as median (IQR) or n (%) as appropriate.

ICU denotes intensive care unit.

⁎ The NEWS2 score is calculated from the following physiological parameters: oxygen saturation, systolic blood pressure, pulse rate, level of consciousness or new-onset confusion, disorientation and/or agitation, and temperature (Royal College of Physicians. National Early Warning Score (NEWS) 2: Standardising the assessment of acute-illness severity in the NHS. Updated report of a working party. London: RCP, 2017).

### 3.3 Risk of in-hospital mortality with COVID-19

**Fig. 3:**
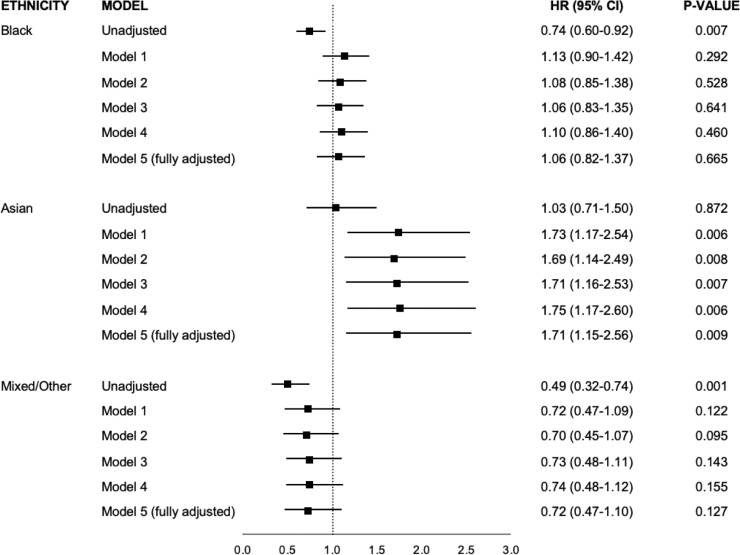
**Association between ethnicity and risk of in-hospital mortality with COVID-19**. Hazard ratios are compared to White ethnicity Model 1 – adjusted for age and sex Model 2 – adjusted for age, sex and index of multiple deprivation Model 3 – adjusted for age, sex and cardiometabolic comorbidities* Model 4 – adjusted for age, sex, and all comorbidities** Model 5 (fully adjusted model) – adjusted for age, sex, index of multiple deprivation, and all comorbidities *Cardiometabolic comorbidities include hypertension, coronary heart disease, heart failure, previous stroke/TIA, diabetes, chronic kidney disease. **Cardiometabolic comorbidities, asthma, chronic obstructive pulmonary disease.

By 2 June 2020, 455 of 1572 patients (28.9%) had died and 1046 (66.5%) had been discharged. The median (IQR) length of stay was 8 (4–16) days. In unadjusted analyses, Black or Mixed/Other ethnicity were associated with a lower risk of death compared to White; however, this was largely due to their younger age (Fig. 3). In age- and sex-adjusted and full adjusted analyses, the risk of death was not statistically significantly different between Black or Mixed/Other ethnicity versus White. By contrast, Asian ethnicity was associated with increased mortality risk which was only slightly attenuated after adjusting for comorbidities or deprivation.

**Fig. 4:**
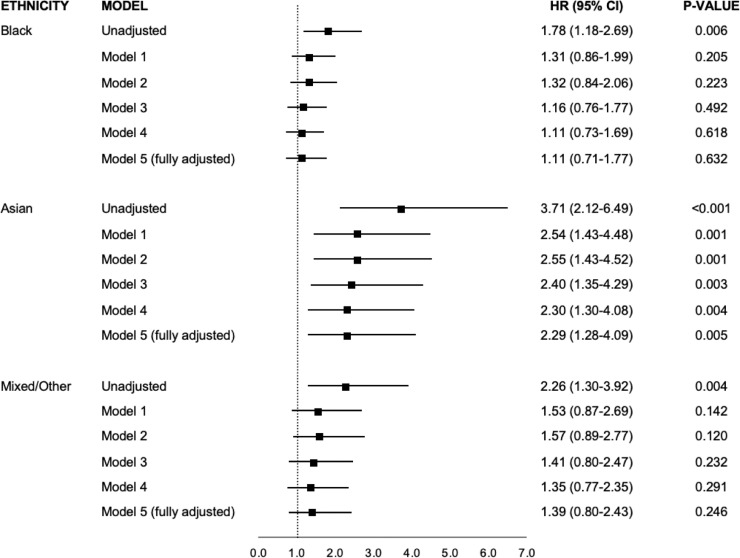
**Association between ethnicity and risk of ICU admission with COVID-19**. Hazard ratios are compared to White ethnicity Model 1 – adjusted for age and sex Model 2 – adjusted for age, sex and index of multiple deprivation Model 3 – adjusted for age, sex and cardiometabolic comorbidities* Model 4 – adjusted for age, sex, and all comorbidities** Model 5 (fully adjusted model) – adjusted for age, sex, index of multiple deprivation, and all comorbidities *Cardiometabolic comorbidities include: hypertension, coronary heart disease, heart failure, previous stroke/TIA, diabetes, chronic kidney disease. **Cardiometabolic comorbidities, asthma, chronic obstructive pulmonary disease.

Asian patients also had statistically significantly higher rates of ICU admission even after adjustment for age, sex, comorbidities and IMD (Fig. 4).

### 3.4 Sensitivity analyses

Sensitivity analyses adjusting for BMI in the subset of patients with BMI available produced results similar to those in the main analysis (Supplemental Fig. 4). Symptom-onset prior to admission was self-reported in 73.2% patients, including a greater proportion of Black and Minority Ethnic than White individuals (Supplemental Table 6). These individuals had higher rates of hypertension and diabetes than patients without a symptom-onset record. Sensitivity analyses in this subset (to examine potential bias due to differential left-censoring) revealed similar findings to the main analysis with respect to mortality (Supplemental Fig. 5). Since the age at hospitalisation was, on average, younger for patients from minority ethnic groups than White patients, the age-specific hazard of death could be different for these groups. To examine potential bias due to left truncation, we performed a sensitivity analysis in patients ≥65 years of age (*n* = 928, 67% White, 22% Black, 5% Asian, 7% Mixed/Other ethnicity). The findings in this subset were similar to those of the main analysis (Supplemental Fig. 6). Finally, using multiple imputation to impute missing values of comorbidity and IMD variables (<5%), fully adjusted hazard ratios for in-hospital mortality were as follows: Black 0.83 (0.62–1.09), Asian 1.55 (0.98–2.45) and Mixed/Other ethnicity 0.67 (0.42–1.07)..

## 4 Discussion

People of Black and other minority ethnic background are reported to have a disproportionately high mortality from COVID-19 both in the US and the UK [5], [6], [7], [8], [9], [10], [11]. However, it is unclear to what extent this is a reflection of the higher proportion of minority ethnic groups in the areas (typically cities) most affected by the pandemic, higher prevalence of cardiovascular, metabolic and other comorbidities, greater socioeconomic deprivation, or other underlying factors. It is also not established whether ethnicity predominantly impacts on the risk of infection, progression of disease once infected, or survival after admission with severe COVID-19.

The current study makes several new findings. First, we employ a case-control study design to identify an approximately 3-fold higher risk of hospital admission with COVID-19 for Black and Mixed ethnicity individuals, but not Asians, as compared to White inner city residents in the same region. Part of the increased risk may relate to comorbidities and deprivation, but there remains a 2.2 to 2.7-fold higher admission risk after adjusting for these factors.

Secondly, we find marked inter-ethnic variation in demographics and comorbidities among admitted patients despite broadly similar clinical severity at presentation. Minority ethnic group patients are on average 10–15 years younger than White patients yet have a higher prevalence of comorbidities. Diabetes is especially prevalent in all non-white groups, consistent with previous community-based data [26,27], while Black patients also have high rates of hypertension and CKD.

Thirdly, we find no association between Black or Mixed ethnicity and in-hospital outcome but an association with increased in-hospital mortality and ICU admission is found for Asian patients. The numbers of Asians in our study is small and they were predominantly non-East Asian, but the association with increased mortality is consistent with a recent UK ICU audit report [7] and preprint data [28]. Overall, our results suggest that Black or Mixed ethnicity are associated with COVID-19 at a different stage in its natural history as compared to Asian ethnicity (i.e. progression to severe symptomatic disease requiring admission versus in-hospital death). Adjusting for cardiometabolic and vascular comorbidities attenuates part of the higher risk but additional ethnicity-related factors may play a large role.

Disproportionately higher admission of Black and Mixed ethnicity individuals compared to White people living in the same region may be driven either by an increased risk of infection, more aggressive early disease progression to the stage requiring admission, or both. An increased susceptibility to infection could occur for several reasons. It has been suggested that a higher prevalence of socioeconomic deprivation (e.g. with poor housing), cultural factors such as living in multi-generational households, and working in higher-risk occupations may increase susceptibility in minority ethnic groups [5]. Adjustment for deprivation as assessed by IMD quintile had only a modest effect in our study. However, it is recognised that the complexity of disadvantage related to socioeconomic factors may be incompletely captured by the IMD score and that such metrics may not be directly comparable across ethnic groups [29]. A recent UK primary care-based study on people who underwent testing for SARS-CoV2 found that the odds of a positive test were statistically significantly higher in Black compared to White individuals [30]. However, the number of Black individuals in this study was <10% and there was no information on disease requiring hospital admission. Interestingly, this study found no association between positive SARS-CoV2 tests and household size. Another UK study in hospital staff who underwent SARS-CoV2 testing found no association between minority ethnic background or frontline working and positive tests [31].

A higher prevalence of comorbidities, especially diabetes, may also increase susceptibility to infection. Furthermore, the impact of diabetes may be disproportionate in minority ethnic groups. For example, diabetes is more likely to progress to complications in African Americans, African Caribbeans and South Asians than White individuals [27,32].

An alternative contributor to higher admission risk in Black and Mixed ethnicity people may be that they are more likely once infected with SARS-CoV2 to progress to disease that requires admission (also considering that an increase in mild infections per se would not necessarily affect admission risk or mortality). Consistent with this idea, it is notable that the ethnicity distribution for COVID-19 admissions in our study was markedly different from that for admissions with respiratory infections in the same community population. Comparisons of rates of hospitalisation with severe COVID-19 for Black versus White patients in the US also suggest higher than expected hospitalisations in the Black group, although data were aggregated across entire states [9,33]. Interestingly, immune responses to pathogens are known to be significantly different between individuals of African versus European ancestry, with African ancestry associated with a stronger inflammatory response [34,35]. Additionally, a higher prevalence of comorbidities such as diabetes could augment pro-inflammatory effects and further promote progression to severe disease. It may be relevant in this context that minority ethnic group patients were much younger than White patients whereas younger age is normally associated with a lower risk of severe disease. Our study was, however, not designed to distinguish between infection risk and more severe early disease as drivers of COVID-19 admission.

We did not find evidence that in-hospital survival was statistically significantly different between Black or Mixed ethnicity and White patients, suggesting that there are no major differences between these groups in life-threatening complications. The age-adjusted incidence of ICU admission was also similar for Black and White patients. However, patients of Asian descent appeared to have strikingly higher in-hospital death and ICU admission than the other ethnic groups. Although the numbers of Asian patients in our study was low and they were predominantly non-East Asian, these results are consistent with other recent reports [7,28] and therefore likely to represent a genuine difference with other minority ethnic groups. We observed a longer symptom duration prior to admission in Asians compared to the other groups but clinical severity at admission as assessed by NEWS2 score was not statistically significantly worse. Whether the longer symptom duration reflects difficulties in access to healthcare or differences in health-seeking behaviours requires further investigation. Possible reasons underlying a higher likelihood of life-threatening complications after admission with COVID-19 in Asian patients also require further study, including assessment of the different Asian sub-categories. It is of interest that African Caribbeans and South Asians with similar degrees of diabetes or metabolic abnormality have quite different profiles of incident cardiovascular disease, with the South Asians much more likely to develop heart disease [36].

Most previous studies that addressed the relationship between ethnicity and COVID-19 either focused solely on mortality referenced to populations aggregated over large geographical areas or made causal inferences from investigation of hospitalised patients alone [[7], [8], [9],^11^,^28^,^33^]. The limitations of these approaches in terms of selection or collider bias have been discussed [37]. A particular issue is the use of reference community populations aggregated over large geographical regions whereas the incidence of severe COVID-19 is typically highly heterogeneous within such regions. For example, a recent very large cohort study not specifically addressing ethnicity and focused solely on mortality, has the significant limitation that the analyses did not take into account contextual local-level socio-demographic and comorbidity profiles [11]. In the present study, the use of a case-control design allowed us to compare in detail the characteristics of admitted patients with a representative sample of the source population and thereby minimise selection bias. Our primary care database covered a large inner city region that closely matched the normal catchment area for the hospital while at the same time enabling individual-level assessment of factors such as comorbidities and deprivation. Our findings are unlikely to be explained by a lower threshold for admission of Black individuals because clinical severity was broadly similar (or slightly worse) for these patients. It is also unlikely that White patients were differentially admitted to other hospitals in the area since emergency admissions are typically to the nearest hospital in the UK National Health Service. Moreover, we present data for all five hospitals in south-east London, which confirm excess COVID-19 admissions in the Black ethnic group. This approach strengthens our conclusions regarding the interrelationship between ethnicity, comorbidities, deprivation score and risk of admission.

Our study was performed in a single multi-ethnic region of inner London to optimise the case-control design involving the primary care database for the region. However, 14 of the 33 London Boroughs have an ethnic minority population of >40% (similar to our region) and 27 have at least 25% ethnic minorities; the overall average for London is approximately 40% non-White [12]. The deprivation profiles for London Boroughs with a high proportion of ethnic minority people are also quite similar. We therefore believe that the results of this study are very likely to be applicable across the whole of London. Emerging reports from other UK cities with high ethnic minority populations also indicate a greater disease burden in the minority groups [38]. Whether these results translate to large cosmopolitan cities with multi-ethnic populations in other countries requires further studies. However, a recent report from New York found markedly higher rates of COVID-19 hospitalisation and death in the Bronx Borough - which has the highest proportion of ethnic minorities - than the other 4 boroughs [39].

Our study has several limitations. We only studied patients who required admission for severe COVID-19. In common with most published studies so far, we did not have data on the exposure to SARS-CoV2 in the community nor on the prevalence of asymptomatic or mild disease. Our conclusions regarding risk of infection are therefore potentially biased by this unknown factor. A small proportion of our cohort had missing ethnicity, consistent with other similar studies. While deriving information on deprivation at individual postcode-level was a strength, we did not have individual-level data on factors such as number of people in the household, occupation, availability of personal protective material, and adherence to social distancing measures, all of which may impact on exposure risk and are potential confounding factors. Information on morbidities such as sleep apnoea and dementia was unavailable nor did we have detailed information on the degree of control of comorbidities or organ function. Therefore, “adjustment” for these factors may not fully account for the associated risk. Conclusions regarding obesity were limited by the high proportion of patients of all ethnic groups for whom data on BMI was unavailable. However, we performed sensitivity analyses to exclude the possibility that our conclusions were significantly confounded by these missing data. The ethnic categories used in our study were broad categories and the number of Asian patients was low; the analyses therefore do not explore variation within subgroups. Finally, the study analysed associations whereas specific mechanisms that may underlie ethnicity-associated risk require confirmation in further investigation.

Despite these limitations, the current findings of increased risk of admission related to Black and Mixed ethnicity versus increased risk of in-hospital mortality related to Asian ethnicity provide new insights into the different ways in which ethnic background impacts on COVID-19 disease trajectory. Our study supports an important contribution of cardiometabolic/vascular factors (notably hypertension and diabetes) to ethnicity-related risk but also suggests a large role for other as yet unidentified factors, whether latent socioeconomic or biological. An intriguing possibility is that specific risks or mechanisms relevant at different stages during the natural history of COVID-19 may be affected to different extents in people of Black versus Asian ethnic background. The identification of such effects is an important area for continuing research. In the meantime, ethnic background may be considered a risk factor for susceptibility to severe COVID-19, in addition to older age, male sex and the presence of cardiometabolic/vascular comorbidities.

## 5 Funding

This work was supported in part by the British Heart Foundation (CH/1999001/11735 and RE/18/2/34213 to AMS) and the National Institute for Health Research Biomedical Research Centres (NIHR BRCs) at Guy's & St Thomas’ NHS Foundation Trust (IS-BRC-1215–20006) and South London and Maudsley NHS Foundation Trust (SLAM; IS-BRC-1215–20018) both with King's College London. AMS is also supported by the Fondation Leducq. RJBD is also supported by Health Data Research UK (HDRUK); UK Research and Innovation (UKRI) London Medical Imaging & Artificial Intelligence Centre for Value Based Healthcare; the BigData@Heart Consortium (Grant No. 116,074 of the European Union Horizon 2020 programme); the NIHR BRC and Research Informatics Unit at University College London Hospitals; and the NIHR Applied Research Collaboration South London at KCHFT. RZ is supported by a King's Prize Fellowship. RB is supported by a Medical Research Council (MRC) Skills Development Fellowship programme (MR/R016372/1) and the NIHR SLAM BRC. DMB holds a UKRI Fellowship as part of HDRUK MR/S00310X/1. KO'G is supported by a MRC Clinical Training Fellowship. The views expressed are those of the authors and not necessarily those of NIHR or the Department of Health and Social Care. The funders had no role in study design, data collection and analysis, decision to publish, or preparation of the manuscript.

## Contributors

JTHT, JG, RJBD and AMS conceived the study. RZ, MA, DMB, SN and MG participated in study design. RZ, HD, SD, KOG, CP, VC, EA, WB, RDB, JTHT and JG participated in data collection. RZ, RB, DMB and SN did data analyses. RZ, RB, MA, WB, RDB, SN, MG, JTHT, JG, RJDB and AMS contributed to data interpretation. RZ and AMS drafted the first version of the manuscript. All authors contributed to and approved the final manuscript and decision to submit. The corresponding author attests that all listed authors meet authorship criteria and that no others meeting the criteria have been omitted.

## Declaration of Competing Interest

JTHT received research funding from Innovate UK & Office of Life Sciences, and iRhythm Technologies, and holds shares <£5000 in Glaxo Smithkline and Biogen. The other authors declare no competing interests.

## Appendix Supplementary materials

Image, application 1

Image, application 2

Availability of data and materials The authors declare that all data supporting the findings of this study are available within the article (and its supplementary information files). Individual participant data will not be made available. Image, application 3
