# Supplementary material for: A case-control and cohort study to determine the relationship between ethnic background and severe COVID-19
Source: eClinicalMedicine. 2020 Oct 9;28:100574. doi: 10.1016/j.eclinm.2020.100574 (PMC7545271; doi:10.1016/j.eclinm.2020.100574)
Supplement: Supplementary file 1 [file mmc1.docx]

**SUPPLEMENTAL MATERIALS**

**Contents**

|  |  | Page |
| --- | --- | --- |
| Methods | Sample size considerations | 2 |
| Tables |  |  |
| Supplemental Table 1 | Characteristics of patients with and without ethnicity data available in the community sample. | 3 |
| Supplemental Table 2 | Characteristics of cases and age-sex-matched population controls stratified by ethnicity | 4 |
| Supplemental Table 3 | Association between ethnicity and risk of COVID-19 admission (Black groups disaggregated). | 5 |
| Supplemental Table 4 | Characteristics of patients with and without ethnicity data available admitted to hospital with COVID-19. | 7 |
| Supplemental Table 5 | Patient characteristics over time (sequential cohorts) by ethnic group | 8 |
| Supplemental Table 6 | Characteristics of patients with and without self-reported symptom-onset date reported. | 9 |
| Figures |  |  |
| Supplemental Figure 1 | Age distribution of patients admitted to hospital with COVID-19 from Inner South East London and community residents by ethnic group | 10 |
| Supplemental Figure 2 | Association between ethnicity and risk of COVID-19 admission in the subset of patients with BMI data available | 11 |
| Supplemental Figure 3 | Ethnicity distribution of emergency hospital admissions in Inner South East London. | 12 |
| Supplemental Figure 4 | Association between ethnicity and risk of in-hospital mortality with COVID-19 in patients with BMI data available | 13 |
| Supplemental Figure 5 | Association between ethnicity and risk of in-hospital mortality with COVID-19 from the date of symptom-onset | 14 |
| Supplemental Figure 6 | Association between ethnicity and risk of in-hospital mortality with COVID-19 among patients >65 years of age only (n=928) | 15 |

**Supplemental Methods**

*Sample size considerations*

Since this study is descriptive and predominantly hypothesis-generating, formal power calculations were not performed a priori. However, to give an idea of precision, for a matched case-control study design, assuming that all patients in the community are at risk of infection with COVID-19, and based on a prevalence of 27.1% Black ethnicity among population controls, a minimum sample of 445 patients with four controls per case (i.e. 89 cases and 356 controls) would provide 80% power, and a minimum sample size of 600 patients (i.e. 120 cases and 480 controls) would provide 90% power, to detect a minimum odds ratio of 2.0 for hospitalisation with COVID-19 associated with Black ethnicity, assuming a 2-sided type I error rate (α) of 0.05 and 90% level of power.

**Supplemental Table 1. Characteristics of patients with and without ethnicity data available in the community sample.**

|  | Etnicity known  N=303,113  (88.1%) | ethnicity unknown  N=40,970  (11.9%) | p-value |
| --- | --- | --- | --- |
|  |  |  |  |
| Demographics |  |  |  |
| Age, years | 38 (29-51) | 35 (27-49) | <0.001 |
| Age, n (%) |  |  |  |
| 18-24 | 26,812 (8.9) | 7,093 (17.3) |  |
| 25-34 | 98,998 (32.7) | 12,860 (31.4) |  |
| 35-44 | 68,487 (22.6) | 8,187 (20.0) |  |
| 45-54 | 47,673 (15.7) | 6,038 (14.7) |  |
| 55-64 | 32,473 (10.7) | 4,192 (10.2) |  |
| 65-74 | 16,304 (5.4) | 1,671 (4.1) |  |
| 75-84 | 8,817 (2.9) | 657 (1.6) |  |
| 85+ | 3,549 (1.2) | 272 (0.7) |  |
| Male sex | 149,305 (49.3) | 24,231 (59.1) | <0.001 |
| Ethnicity |  |  |  |
| White | 189,443 (62.5) | - |  |
| Black | 62,073 (20.5) | - |  |
| Asian | 24,082 (7.9) | - |  |
| Mixed/Other | 27,515 (9.1) | - |  |
|  |  |  |  |
| Comorbidities |  |  |  |
| Cardiovascular |  |  |  |
| Hypertension | 35,527 (11.7) | 2,318 (5.7) | <0.001 |
| Coronary heart disease | 4,635 (1.5) | 259 (0.6) | <0.001 |
| Heart failure | 1,769 (0.6) | 106 (0.3) | <0.001 |
| Previous stroke/TIA | 3,413 (1.1) | 251 (0.6) | <0.001 |
| Metabolic |  |  |  |
| Diabetes | 20,081 (6.6) | 1,223 (3.0) | <0.001 |
| Chronic kidney disease | 5,955 (2.0) | 328 (0.8) | <0.001 |
| Other |  |  |  |
| Asthma | 13,683 (4.5) | 953 (2.3) | <0.001 |
| COPD | 3,572 (1.2) | 146 (0.4) | <0.001 |
|  |  |  |  |
| Socioeconomic factors |  |  |  |
| Deprivation quintile |  |  | <0.001 |
| 1 (most deprived) | 55,495 (18.3) | 8,175 (20.0) |  |
| 2 | 139,647 (46.1) | 19,233 (46.9) |  |
| 3 | 80,375 (26.5) | 10,124 (24.7) |  |
| 4 | 20,831 (6.9) | 2,650 (6.5) |  |
| 5 (least deprived) | 3,675 (1.2) | 391 (1.0) |  |
| Missing | 3,090 (1.0) | 397 (1.0) |  |

Data are presented as n (%) or median (IQR) as appropriate

**Supplemental Table 2. Characteristics of COVID-19 cases and age-sex-matched population controls stratified by ethnicity**

|  | White | | Black | | Asian | | Mixed/Other | |
| --- | --- | --- | --- | --- | --- | --- | --- | --- |
|  | Cases  N=294 | Controls  N=2,019 | Cases  N=419 | Controls  N=944 | Cases  N=49 | Controls  N=273 | Cases  N=110 | Controls  N=252 |
| Comorbidities |  |  |  |  |  |  |  |  |
| Cardiovascular |  |  |  |  |  |  |  |  |
| Hypertension | 200 (68.0) | 714 (35.4) | 305 (72.8) | 537 (56.9) | 32 (65.3) | 120 (44.0) | 57 (51.8) | 90 (35.7) |
| Coronary heart disease | 64 (21.8) | 174 (8.6) | 57 (13.6) | 60 (6.4) | 9 (18.4) | 29 (10.6) | 19 (17.3) | 12 (4.8) |
| Heart failure | 48 (16.3) | 70 (3.5) | 66 (15.8) | 44 (4.7) | 9 (18.4) | 6 (2.2) | 14 (12.7) | 3 (1.2) |
| Previous stroke/TIA | 64 (21.8) | 116 (5.8) | 66 (15.8) | 69 (7.3) | 8 (16.3) | 16 (5.9) | 8 (7.3) | 12 (4.8) |
| Metabolic |  |  |  |  |  |  |  |  |
| Diabetes | 97 (33.0) | 255 (12.6) | 222 (53.0) | 275 (29.1) | 27 (55.1) | 91 (33.3) | 43 (39.1) | 48 (19.1) |
| Chronic kidney disease | 68 (23.1) | 202 (10.0) | 129 (30.8) | 128 (13.6) | 13 (26.5) | 33 (12.1) | 18 (16.4) | 17 (6.8) |
| Other |  |  |  |  |  |  |  |  |
| Asthma | 34 (11.6) | 129 (6.4) | 67 (16.0) | 68 (7.2) | 7 (14.3) | 28 (10.3) | 15 (13.6) | 18 (7.1) |
| COPD | 62 (21.1) | 152 (7.5) | 27 (6.4) | 30 (3.2) | 4 (8.2) | 10 (3.7) | 10 (9.1) | 8 (3.2) |
| Multimorbidity* | 182 (61.9) | 492 (24.4) | 277 (66.1) | 359 (38.0) | 31 (63.3) | 102 (37.4) | 54 (49.1) | 63 (25.0) |
|  |  |  |  |  |  |  |  |  |
| BMI |  |  |  |  |  |  |  |  |
| Underweight | 19 (6.5) | 63 (3.1) | 6 (1.4) | 16 (1.7) | 1 (2.0) | 11 (4.0) | 3 (2.7) | 6 (2.4) |
| Normal weight | 76 (25.9) | 789 (39.1) | 54 (12.9) | 223 (23.6) | 3 (6.1) | 51 (18.7) | 17 (15.5) | 73 (29.0) |
| Overweight | 60 (20.4) | 648 (32.1) | 82 (19.6) | 340 (36.0) | 8 (16.3) | 112 (41.0) | 17 (15.5) | 79 (31.3) |
| Obese | 51 (17.3) | 396 (19.6) | 116 (27.7) | 322 (34.1) | 14 (28.6) | 77 (28.2) | 22 (20.0) | 64 (25.4) |
| Missing | 88 (29.9) | 123 (6.1) | 161 (38.4) | 43 (4.6) | 23 (46.9) | 22 (8.1) | 51 (46.4) | 30 (11.9) |
|  |  |  |  |  |  |  |  |  |
| Socioeconomic factors |  |  |  |  |  |  |  |  |
| IMD quintile |  |  |  |  |  |  |  |  |
| 1 (most deprived) | 74 (25.2) | 302 (15.0) | 139 (33.2) | 278 (29.4) | 12 (24.5) | 34 (12.5) | 30 (27.3) | 64 (25.4) |
| 2 | 127 (43.2) | 885 (43.8) | 186 (44.4) | 469 (49.7) | 26 (53.1) | 105 (38.5) | 53 (48.2) | 115 (45.6) |
| 3 | 64 (21.8) | 631 (31.3) | 80 (19.1) | 164 (17.4) | 10 (20.4) | 98 (35.9) | 23 (20.9) | 60 (23.8) |
| 4 | 27 (9.2) | 161 (8.0) | 11 (2.6) | 28 (3.0) | 1 (2.0) | 34 (12.5) | 4 (3.6) | 13 (5.2) |
| 5 (least deprived) | 1 (0.3) | 27 (1.3) | 2 (0.5) | 2 (0.2) | 0 | 2 (0.7) | 0 | 0 |
| Missing | 1 (0.3) | 13 (0.6) | 1 (0.2) | 3 (0.3) | - | - | - | - |

Data presented as n (%). BMI denotes body mass index; COPD, chronic obstructive pulmonary disease; IMD, index of multiple deprivation

* Defined as ≥2 comorbidities

**Supplemental Table 3. Association between ethnicity and risk of COVID-19 admission (Black groups disaggregated).**

| Ethnicity | Model | OR (95% CI) | p-value |
| --- | --- | --- | --- |
| Black African | Unadjusted | 4.73 (3.82-5.85) | <0.001 |
|  | Model 1 | 4.73 (3.82-5.85) | <0.001 |
|  | Model 2 | 4.37 (3.52-5.43) | <0.001 |
|  | Model 3 | 3.50 (2.75-4.45) | <0.001 |
|  | Model 4 | 3.81 (2.98-4.87) | <0.001 |
|  | Model 5 (fully adjusted) | 3.66 (2.85-4.70) | <0.001 |
|  |  |  |  |
| Black Caribbean | Unadjusted | 2.09 (1.67-2.62) | <0.001 |
|  | Model 1 | 2.09 (1.67-2.62) | <0.001 |
|  | Model 2 | 1.96 (1.56-2.47) | <0.001 |
|  | Model 3 | 1.42 (1.10-1.82) | 0.007 |
|  | Model 4 | 1.49 (1.15-1.92) | 0.002 |
|  | Model 5 (fully adjusted) | 1.44 (1.11-1.86) | 0.006 |
|  |  |  |  |
| Black Other | Unadjusted | 2.52 (1.67-3.81) | <0.001 |
|  | Model 1 | 2.53 (1.67-3.81) | <0.001 |
|  | Model 2 | 2.37 (1.57-3.58) | <0.001 |
|  | Model 3 | 1.71 (1.08-2.72) | 0.023 |
|  | Model 4 | 1.78 (1.12-2.85) | 0.016 |
|  | Model 5 (fully adjusted) | 1.72 (1.07-2.75) | 0.025 |
|  |  |  |  |
| Asian | Unadjusted | 1.20 (0.86-1.66) | 0.288 |
|  | Model 1 | 1.20 (0.86-1.66) | 0.288 |
|  | Model 2 | 1.20 (0.86-1.67) | 0.277 |
|  | Model 3 | 0.96 (0.66-1.37) | 0.805 |
|  | Model 4 | 1.01 (0.70-1.45) | 0.977 |
|  | Model 5 (fully adjusted) | 1.02 (0.70-1.47) | 0.936 |
|  |  |  |  |
| Mixed/Other | Unadjusted | 3.00 (2.32-3.89) | <0.001 |
|  | Model 1 | 3.00 (2.32-3.89) | <0.001 |
|  | Model 2 | 2.84 (2.19-3.68) | <0.001 |
|  | Model 3 | 2.75 (2.07-3.65) | <0.001 |
|  | Model 4 | 2.84 (2.13-3.78) | <0.001 |
|  | Model 5 (fully adjusted) | 2.75 (2.06-3.66) | <0.001 |

Odds ratios are compared to White ethnicity

Model 1 – adjusted for age and sex

Model 2 – adjusted for age, sex and index of multiple deprivation

Model 3 – adjusted for age, sex and cardiometabolic comorbidities*

Model 4 – adjusted for age, sex, and all comorbidities**

Model 5 (fully adjusted model) – adjusted for age, sex, index of multiple deprivation, and all comorbidities

*Cardiometabolic comorbidities include hypertension, coronary heart disease, heart failure, previous stroke/TIA, diabetes, chronic kidney disease.

** Cardiometabolic comorbidities, asthma, chronic obstructive pulmonary disease.

Black African n=238; Black Caribbean n=145; Other Black n=36

**Supplemental Table 4. Characteristics of patients with and without ethnicity data available admitted to hospital with COVID-19.**

|  | Etnicity KNown  N=1,572  (86.0%) | ethnicity unknown  N=255  (14.0%) | p-value |
| --- | --- | --- | --- |
|  |  |  |  |
| Demographics |  |  |  |
| Age, years | 70 (56-82) | 61 (50-75) | <0.001 |
| Age, n (%) |  |  |  |
| 18-24 | 17 (1.1) | 5 (2.0) | - |
| 25-34 | 52 (3.3) | 14 (5.5) | - |
| 35-44 | 92 (5.9) | 22 (8.6) | - |
| 45-54 | 201 (12.8) | 45 (17.7) | - |
| 55-64 | 282 (17.9) | 53 (20.8) | - |
| 65-74 | 279 (17.8) | 47 (18.4) | - |
| 75-84 | 345 (22.0) | 36 (14.1) | - |
| 85+ | 304 (19.3) | 33 (12.9) | - |
| Male sex | 886 (56.4) | 158 (62.0) | 0.094 |
| Ethnicity |  |  |  |
| White | 845 (53.8) | - |  |
| Black | 486 (30.9) | - |  |
| Asian | 90 (5.7) | - |  |
| Mixed/Other | 151 (9.6) | - |  |
|  |  |  |  |
| Comorbidities |  |  |  |
| Cardiovascular |  |  |  |
| Hypertension | 1,027 (65.3) | 120 (47.2) | <0.001 |
| Coronary heart disease | 288 (18.3) | 20 (7.9) | <0.001 |
| Heart failure | 313 (20.0) | 21 (8.3) | <0.001 |
| Previous stroke/TIA | 355 (22.6) | 24 (9.5) | <0.001 |
| Metabolic |  |  |  |
| Diabetes | 594 (37.8) | 79 (31.1) | 0.040 |
| Chronic kidney disease | 405 (25.8) | 37 (14.6) | <0.001 |
| Other |  |  |  |
| Asthma | 193 (12.3) | 18 (7.1) | 0.016 |
| COPD | 202 (12.9) | 16 (6.3) | 0.003 |
|  |  |  |  |
| Socioeconomic factors |  |  |  |
| Deprivation quintile |  |  | 0.462 |
| 1 (most deprived) | 331 (21.1) | 52 (20.4) |  |
| 2 | 487 (31.0) | 71 (27.8) |  |
| 3 | 280 (17.8) | 45 (17.6) |  |
| 4 | 239 (15.2) | 50 (19.6) |  |
| 5 (least deprived) | 225 (14.3) | 34 (13.3) |  |
| Missing | 10 (0.6) | 3 (1.2) |  |

Data are presented as n (%) or median (IQR) as appropriate

**Supplemental Table 5. Patient characteristics over time (sequential cohorts) by ethnic group**

| cohort | 1  N=250 | 2  N=250 | 3  N=250 | 4  N=250 | 5  N=250 | 6  N=322 | p-value |
| --- | --- | --- | --- | --- | --- | --- | --- |
| Age, years |  |  |  |  |  |  |  |
| White | 75 (63-85) | 74 (64-84) | 73 (59-84) | 77 (64-86) | 77 (65-87) | 79 (64-87) | 0.147 |
| Black | 58 (49-70) | 58 (50-71) | 63 (52-76) | 63 (55-76) | 65 (55-76) | 64 (52-78) | 0.156 |
| Asian | 70 (43-77) | 53 (45-69) | 59 (48-74) | 60 (43-75) | 69 (50-78) | 59 (45-80) | 0.873 |
| Mixed/Other | 55 (45-72) | 56 (47-68) | 55 (42-78) | 57 (49-77) | 63 (51-79) | 66 (52-78) | 0.376 |
| Male sex |  |  |  |  |  |  |  |
| White | 59 (51.8) | 82 (63.1) | 79 (59.9) | 68 (52.3) | 72 (58.5) | 116 (53.7) | 0.332 |
| Black | 46 (45.5) | 46 (52.3) | 44 (52.4) | 44 (62.9) | 55 (70.5) | 34 (52.3) | 0.018 |
| Asian | 6 (42.9) | 8 (88.9) | 5 (45.5) | 18 (69.2) | 9 (56.3) | 6 (42.9) | 0.144 |
| Mixed/Other | 14 (66.7) | 15 (65.2) | 9 (39.1) | 16 (66.7) | 20 (60.6) | 15 (55.6) | 0.371 |

Cohort 1 refers to the first 250 patients admitted, cohort 2 refers to the second 250 patients, and so on.

**Supplemental Table 6. Characteristics of patients with and without self-reported symptom-onset date reported.**

|  | symptom-onset not reported  N=421  (26.8%) | symptom-onset  reported  N=1,151  (73.2%) | p-value |
| --- | --- | --- | --- |
|  |  |  |  |
| Demographics |  |  |  |
| Age, years | 68 (54-82) | 71 (56-82) | 0.122 |
| Age, n (%) |  |  | 0.140 |
| 18-24 | 7 (1.7) | 10 (0.9) |  |
| 25-34 | 23 (5.5) | 29 (2.5) |  |
| 35-44 | 26 (6.2) | 66 (5.7) |  |
| 45-54 | 54 (12.8) | 147 (12.8) |  |
| 55-64 | 72 (17.1) | 210 (18.3) |  |
| 65-74 | 75 (17.8) | 204 (17.7) |  |
| 75-84 | 88 (20.9) | 257 (22.3) |  |
| 85+ | 76 (18.1) | 228 (19.8) |  |
| Male sex | 228 (54.2) | 658 (57.2) | 0.286 |
| Ethnicity |  |  | <0.001 |
| White | 265 (63.0) | 580 (50.4) |  |
| Black | 102 (24.2) | 384 (33.4) |  |
| Asian | 19 (4.5) | 71 (6.2) |  |
| Mixed/Other | 35 (8.3) | 116 (10.1) |  |
|  |  |  |  |
| Comorbidities |  |  |  |
| Cardiovascular |  |  |  |
| Hypertension | 245 (58.2) | 782 (67.9) | <0.001 |
| Coronary heart disease | 68 (16.2) | 220 (19.1) | 0.179 |
| Heart failure | 84 (20.1) | 229 (19.9) | 0.930 |
| Previous stroke/TIA | 72 (17.2) | 283 (24.6) | 0.002 |
| Metabolic |  |  |  |
| Diabetes | 115 (27.3) | 479 (41.6) | <0.001 |
| Chronic kidney disease | 101 (24.0) | 304 (26.4) | 0.331 |
| Other |  |  |  |
| Asthma | 32 (7.7) | 161 (14.0) | 0.001 |
| COPD | 50 (11.9) | 152 (13.2) | 0.486 |
|  |  |  |  |
| Socioeconomic factors |  |  |  |
| Deprivation quintile |  |  | 0.473 |
| 1 (most deprived) | 79 (18.8) | 252 (21.9) |  |
| 2 | 126 (29.9) | 361 (31.4) |  |
| 3 | 79 (18.8) | 201 (17.5) |  |
| 4 | 64 (15.2) | 175 (15.2) |  |
| 5 (least deprived) | 69 (16.4) | 156 (13.6) |  |
| Missing | 4 (1.0) | 6 (0.5) |  |

Data are presented as n (%) or median (IQR) as appropriate

**Supplemental Figure 1. Age distribution of patients admitted to hospital with COVID-19 from Inner South East London and community residents by ethnic group.**

p<0.001

p<0.001

p<0.001

p<0.001

The prevalence is the percentage of individuals in the relevant age band for that ethnic group (e.g. 38% of all White adults in the community are aged 25-34). *p*-values refer to χ-square test of independence, testing the null hypothesis that the proportion of patients in each age group is statistically not different between hospital and community cohorts.

**
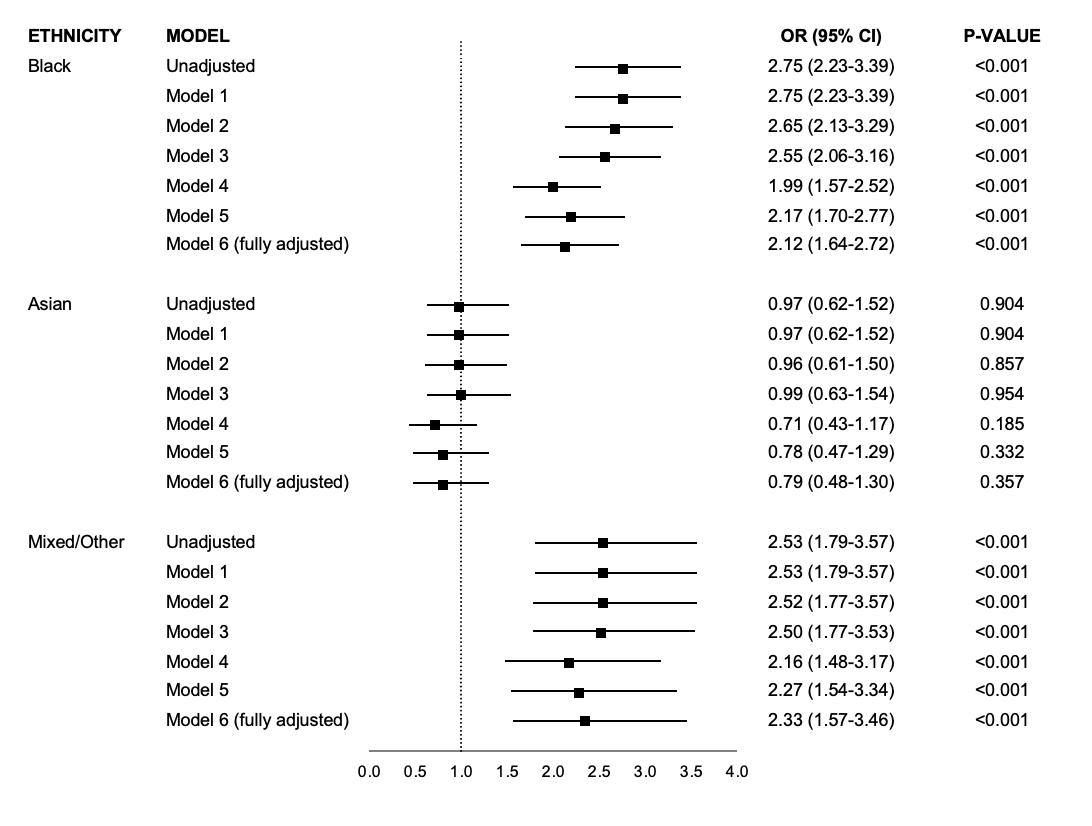
Supplemental Figure 2. Association between ethnicity and risk of COVID-19 admission in the subset of patients with BMI data available**

Odds ratios are compared to White ethnicity

Model 1 – adjusted for age and sex

Model 2 – adjusted for age, sex and index of multiple deprivation (IMD)

Model 3 – adjusted for age, sex and body mass index (BMI)

Model 4 – adjusted for age, sex and cardiometabolic comorbidities*

Model 5 – adjusted for age, sex, and all comorbidities**

Model 6 (fully adjusted model) – adjusted for age, sex, IMD, BMI, and all comorbidities

*Cardiometabolic comorbidities include hypertension, coronary heart disease, heart failure, previous stroke/TIA, diabetes, chronic kidney disease.

** Cardiometabolic comorbidities, asthma, chronic obstructive pulmonary disease.

**Supplemental Figure 3. Ethnicity distribution of emergency hospital admissions in Inner South East London.**

Data on pre-COVID-19 era (March 1st 2019 to February 29th 2020) emergency admissions to hospitals within inner SE London (namely KCHFT Denmark Hill, Guy’s & St Thomas’ NHS Foundation Trust, and Lewisham & Woolwich NHS Trust) were retrieved from the Healthcare Evaluation Data system (HED; [www.hed.nhs.uk](https://www.google.com/url?q=http://www.hed.nhs.uk&sa=D&ust=1592384046163000&usg=AFQjCNEyna6Mk0x1XGiVd-f3oU_Q2NKdnw)) on 2nd June 2020. HED facilitates analyses of NHS Digital’s Hospital Episode Statistics (HES) and ONS datasets. Anonymised data on numbers of admissions with COVID-19 and ethnicity breakdown were obtained directly from these three NHS Trusts. These data represent 90-95% of all emergency admission from patients who are resident in this region.

**Supplemental Figure 4. Association between ethnicity and risk of in-hospital mortality with COVID-19 in patients with BMI data available**

**
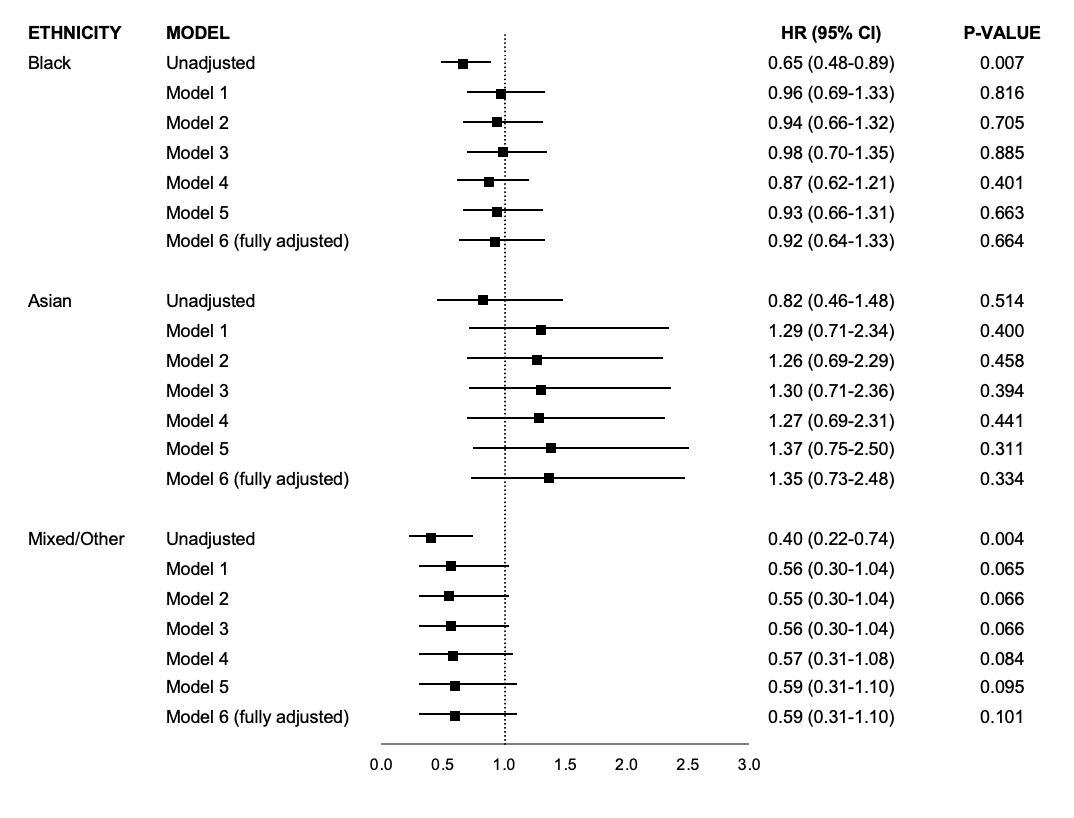
**

Hazard ratios are compared to White ethnicity

Model 1 – adjusted for age and sex

Model 2 – adjusted for age, sex and index of multiple deprivation (IMD)

Model 3 – adjusted for age, sex and body mass index (BMI)

Model 4 – adjusted for age, sex and cardiometabolic comorbidities*

Model 5 – adjusted for age, sex, and all comorbidities**

Model 6 (fully adjusted model) – adjusted for age, sex, IMD, BMI, and all comorbidities

*Cardiometabolic comorbidities include hypertension, coronary heart disease, heart failure, previous stroke/TIA, diabetes, chronic kidney disease.

** Cardiometabolic comorbidities, asthma, chronic obstructive pulmonary disease.

**
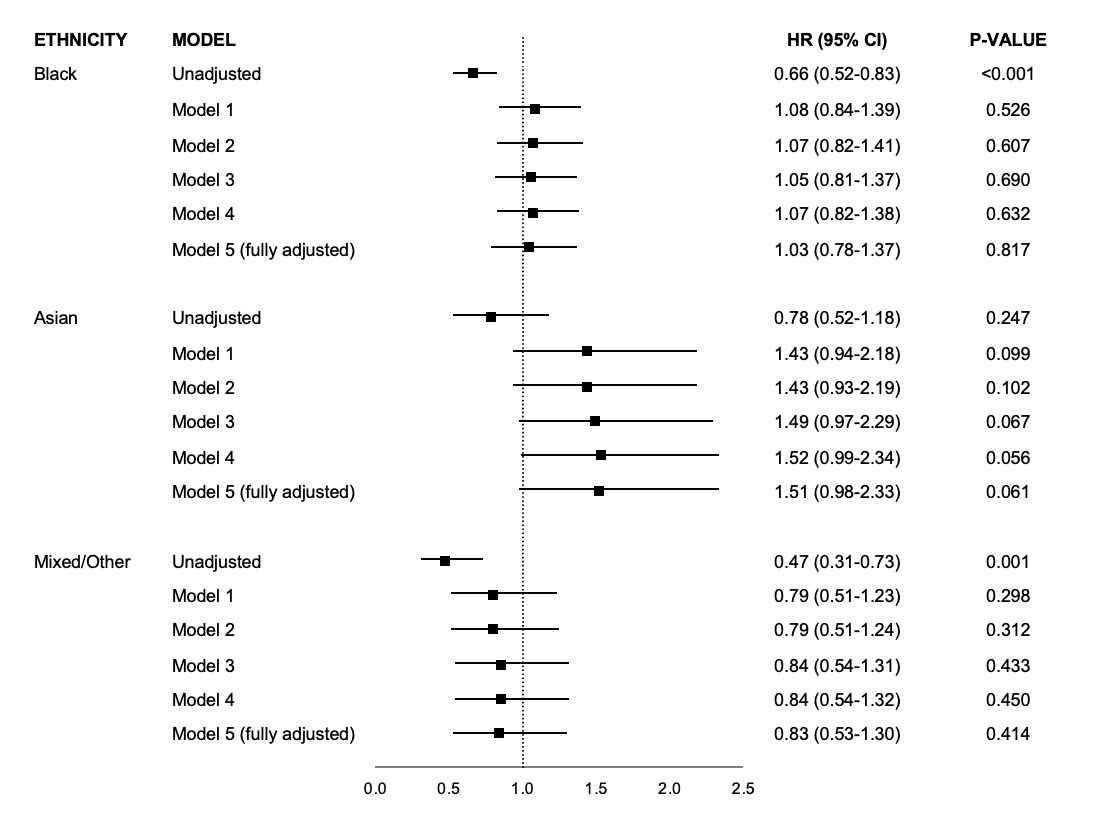
Supplemental Figure 5. Association between ethnicity and risk of in-hospital mortality with COVID-19 from the date of symptom-onset**

Hazard ratios are compared to White ethnicity

Model 1 – adjusted for age and sex

Model 2 – adjusted for age, sex and index of multiple deprivation (IMD)

Model 3 – adjusted for age, sex and body mass index (BMI)

Model 4 – adjusted for age, sex and cardiometabolic comorbidities*

Model 5 – adjusted for age, sex, and all comorbidities**

Model 6 (fully adjusted model) – adjusted for age, sex, IMD, BMI, and all comorbidities

*Cardiometabolic comorbidities include hypertension, coronary heart disease, heart failure, previous stroke/TIA, diabetes, chronic kidney disease.

** Cardiometabolic comorbidities, asthma, chronic obstructive pulmonary disease.

**Supplemental Figure 6. Association between ethnicity and risk of in-hospital mortality with COVID-19 among patients >65 years of age only (n=928)**

**
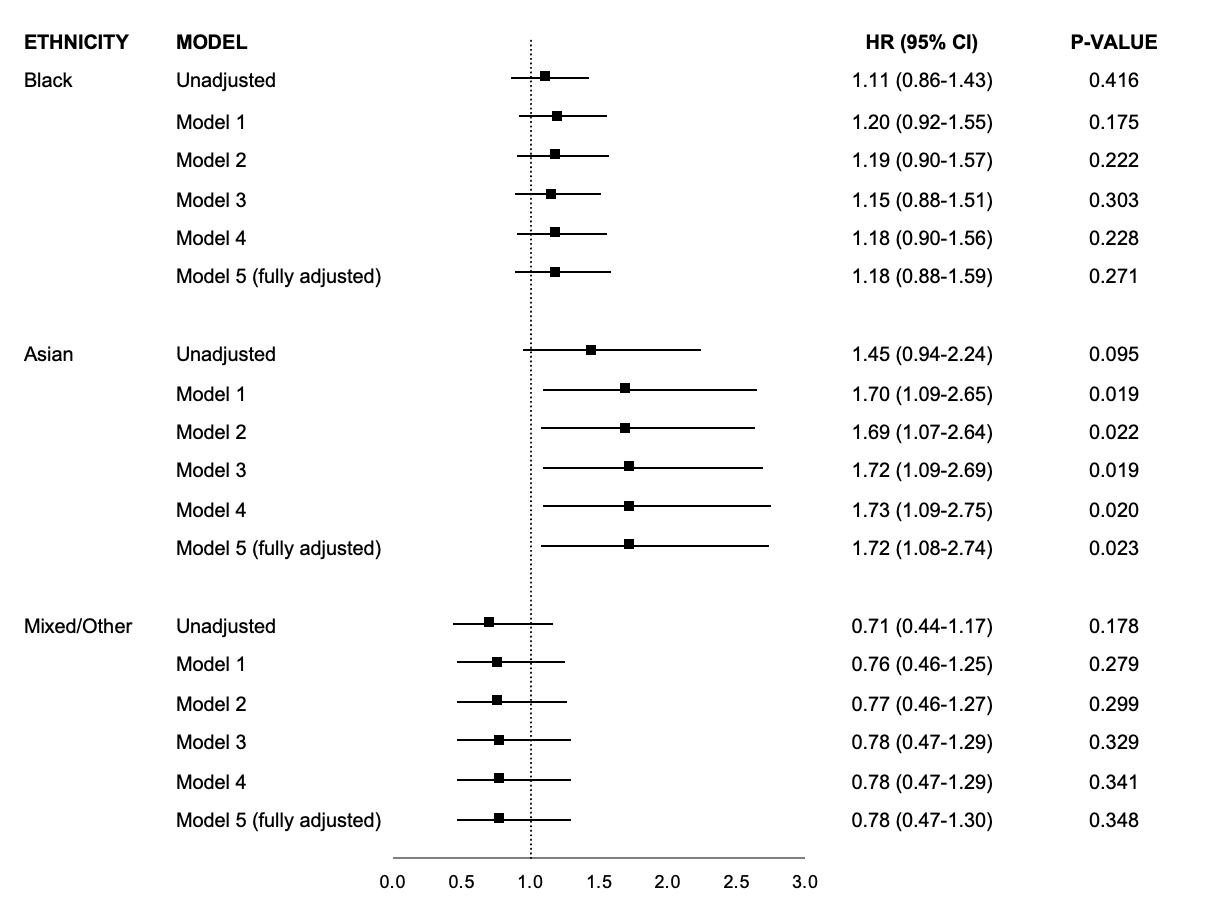
**

Hazard ratios are compared to White ethnicity

Model 1 – adjusted for age and sex

Model 2 – adjusted for age, sex and index of multiple deprivation (IMD)

Model 3 – adjusted for age, sex and body mass index (BMI)

Model 4 – adjusted for age, sex and cardiometabolic comorbidities*

Model 5 – adjusted for age, sex, and all comorbidities**

Model 6 (fully adjusted model) – adjusted for age, sex, IMD, BMI, and all comorbidities

*Cardiometabolic comorbidities include hypertension, coronary heart disease, heart failure, previous stroke/TIA, diabetes, chronic kidney disease.

** Cardiometabolic comorbidities, asthma, chronic obstructive pulmonary disease.
