# Supplementary material for: A case-control and cohort study to determine the relationship between ethnic background and severe COVID-19
Source: eClinicalMedicine. 2020 Oct 9;28:100574. doi: 10.1016/j.eclinm.2020.100574 (PMC7545271; doi:10.1016/j.eclinm.2020.100574)
Supplement: Supplementary file 3 — Availability of data and materials The authors declare that all data supporting the findings of this study are available within the article (and its supplementary information files). Individual participant data will not be made available. [file mmc3.pdf]

15 September, 2020

A case-control and cohort study to determine the relationship between ethnic background and severe COVID-19 by Zakeri et al (Corresponding author Ajay M Shah).

This is to confirm that I am happy to be named in the acknowledgements section of the above manuscript.

Yours sincerely

Professor Clive Kay

15 September, 2020

A case-control and cohort study to determine the relationship between ethnic background and severe COVID-19 by Zakeri et al (Corresponding author Ajay M Shah).

This is to confirm that I am happy to be named in the acknowledgements section of the above manuscript.

Yours sincerely

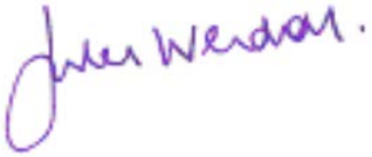

Professor Julia Wendon

15 September, 2020

A case-control and cohort study to determine the relationship between ethnic background and severe COVID-19 by Zakeri et al (Corresponding author Ajay M Shah).

This is to confirm that I am happy to be named in the acknowledgements section of the above manuscript.

Yours sincerely

Professor Irene Higginson

15 September, 2020

A case-control and cohort study to determine the relationship between ethnic background and severe COVID-19 by Zakeri et al (Corresponding author Ajay M Shah).

This is to confirm that I am happy to be named in the acknowledgements section of the above manuscript.

Yours sincerely

Professor Alastair Baker

15 September, 2020

A case-control and cohort study to determine the relationship between ethnic background and severe COVID-19 by Zakeri et al (Corresponding author Ajay M Shah).

This is to confirm that I am happy to be named in the acknowledgements section of the above manuscript.

Yours sincerely

Chris Fry

Dominic Thurgood

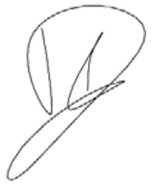

Isuf Ali

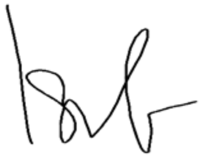

15 September, 2020

A case-control and cohort study to determine the relationship between ethnic background and severe COVID-19 by Zakeri et al (Corresponding author Ajay M Shah).

This is to confirm that I am happy to be named in the acknowledgements section of the above manuscript.

Yours sincerely

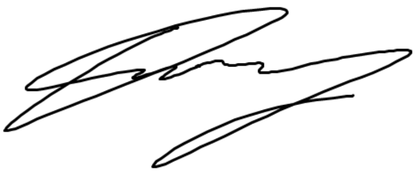

Chris Fry

Dominic Thurgood

Isuf Ali
